# Risks and rewards of AI democratization

**DOI:** 10.1002/ueg2.12560

**Published:** 2024-03-25

**Authors:** Fons van der Sommen, Jeroen de Groof

**Affiliations:** ^1^ VCA Group Eindhoven University of Technology ‐ Department of Electrical Engineering Eindhoven The Netherlands; ^2^ Amsterdam University Medical Centres ‐ Department of Gastroenterology and Hepatology Amsterdam The Netherlands

As the technology driving artificial intelligence (AI) is improving at an unprecedented pace, it has also found its way to a broader audience. Not only in the form of general purpose applications, such as ChatGPT (https://chat.openai.com/) for language or Midjourney (https://www.midjourney.com/home) for image generation, but also ready‐for‐use tools for development are becoming more and more accessible. This allows researchers to learn the basics of AI and readily apply it in their own field, sometimes even without the need for a single line of programming code. This *democratization of AI* substantially increases its potential impact, as it empowers scientists from all disciplines to leverage this disruptive technology for driving progress.

One potent example in the field of GI endoscopy, is the use of AI for disease detection and characterization, where studies have shown that AI can boost detection rates.^1^, ^2^, ^3^ In this issue of the United European Gastroenterology Journal, Gong and colleagues present the results of a study towards a computer‐aided diagnosis (CADx) system for real‐time staging of gastric carcinogenesis. In their experiments, the authors have used a considerable training set, including images of over 11 thousand patients, and demonstrated six‐class classification accuracies of 91.2% and 82.3% on an internal and external data set, respectively. Also noteworthy, is that the authors employed an imbalanced class distribution, that better resembles clinical incidence. This approach leads to more reliable numbers than using largely uniform class distributions, as is often seen in such studies, rendering it harder to gauge clinical applicability of the presented CADx system.

The observed discrepancy between the internal‐ and external test set results, highlights the importance of thorough and careful AI validation, as it reveals some potential biases that have found their way into the experimental design. For example, the internal validation set and internal test set were randomly sampled from the same data set, of which also the training set was drawn. Given that a large number of patients contributed multiple images to this set, images from the same patient may have ended up in both training, validation and/or internal test set, leading to intra‐patient bias. Additionally, the control cases were retrospectively acquired over a different time frame, for which the exact image capturing settings and/or equipment may have been different. It cannot be emphasized enough, how easily modern AI models pick up on such biases. Very slight changes in data that may even be imperceivable to the human eye, may stand out as beacons for image analysis algorithms. It is, therefore, no wonder that multiple tutorials and guidelines have been published on how to reliably train, validate and test supportive AI systems in medicine.^4^, ^5^, ^6^, ^7^

Gong and colleagues leveraged a *no‐code deep‐learning tool* for developing and evaluating the proposed CADx model. As said, on the one hand this allows groups without substantial technical expertise to experiment with AI models for novel applications, while on the other hand also introducing additional risk of bias and hampering reproducibility. Researchers may be unaware of these risks, leading to models that poorly generalize to new data. Robust AI development is not straightforward, demonstrated by an emerging body of work revealing that most models do not live up to their expectations in clinical practice,^8^ including commercial systems that have acquired regulatory clearance.^9^ Also in the experiments of Gong and colleagues, a considerable performance drop was observed when the system was evaluated on an external test set, collected at a different hospital. This lack of generalization may be explained by a phenomenon known as domain shift or ‐gap,^10^ which occurs when the data used to develop the AI system with is different than the data it will encounter during its application. For example, an AI system may be trained and tested with high‐quality data collected at academic centers, while the data it will see in daily clinical practice is more heterogeneous, leading to a significant degradation of AI performance.^11^ Now that an growing number of AI systems are tested in a clinical setting, such domain gaps will become increasingly apparent and will need to be critically appraised from both a clinical *and* technical perspective.

There is definitely a place for tools that allow researchers with little AI expertise perform feasibility experiments for yet unexplored applications. However, it is important that the users of such platforms are informed about the potential risks that may deteriorate the reliability of their results. Multidisciplinary collaborations between clinicians and engineers remain however invaluable in the development of responsible and safe AI systems for clinical decision support.

## CONFLICT OF INTEREST STATEMENT

The authors have no conflicts of interest to declare.

## Data Availability

Data sharing is not applicable to this article as no new data were created or analyzed in this study.
